# Embracing the Nutritional Assessment in Cerebral Palsy: A Toolkit for Healthcare Professionals for Daily Practice

**DOI:** 10.3390/nu14061180

**Published:** 2022-03-11

**Authors:** Carolina Pinto, Rute Borrego, Mafalda Eiró-Gomes, Inês Casimiro, Ana Raposo, Teresa Folha, Daniel Virella, Ana Catarina Moreira

**Affiliations:** 1 Escola Superior de Tecnologia da Saúde de Lisboa-Instituto Politécnico de Lisboa (ESTeSL-IPL), 1990-096 Lisboa, Portugal; rute.borrego@estesl.ipl.pt (R.B.); ana.moreira@estesl.ipl.pt (A.C.M.); 2 Faculdade de Medicina, Universidade de Lisboa (FM-UL), 1649-028 Lisboa, Portugal; 3 Escola Superior de Comunicação Social-Instituto Politécnico de Lisboa, 1549-014 Lisboa, Portugal; agomes@escs.ipl.pt (M.E.-G.); casimiro.ines@gmail.com (I.C.); ana.raposo@escs.ipl.pt (A.R.); 4 Departamento de Epidemiologia, Instituto Nacional de Saúde Doutor Ricardo Jorge (INSA), 1600-609 Lisboa, Portugal; m.teresa.folha@insa.min-saude.pt (T.F.); daniel.virella@insa.min-saude.pt (D.V.); 5 Programa de Vigilância Nacional da Paralisia Cerebral Consortium, 1600-609 Lisboa, Portugal; 6 H&TRC—Health & Technology Research Center, 1990-096 Lisboa, Portugal

**Keywords:** cerebral palsy, anthropometric measures, underreported data, surveillance program, child, neurology

## Abstract

Background: Nutritional status assessment (NSA) can be challenging in children with cerebral palsy (CP). There are high omission rates in national surveillance reports of weight and height information. Alternative methods are used to assess nutritional status that may be unknown to the healthcare professionals (HCP) who report these children. Caregivers experience challenges when dealing with feeding problems (FP) common in CP. Our aim was to assess the difficulties in NSA which are causing this underreport and to create solutions for registers and caregivers. Methods: An online questionnaire was created for registers. Three meetings with HCP and caregivers were held to discuss problems and solutions regarding NSA and intervention. Results: HCP mentioned difficulty in NSA due to a lack of time, collaboration with others, equipment, and childrens’ motor impairment. Caregivers experienced difficulty in preparing nutritious meals with adapted textures. The creation of educational tools and other strategies were suggested. A toolkit for HCP was created with the weight and height assessment methods described and other for caregivers to deal with common FP. Conclusions: There are several difficulties experienced by HCP that might be overcome with educational tools, such as a toolkit. This will facilitate nutritional assessment and intervention and hopefully reduce underreporting.

## 1. Introduction

Cerebral palsy (PC) is associated with motor problems accompanied by disorders in cognition, communication and behavior, epilepsy episodes, and musculoskeletal problems. It is the most common motor deficiency in childhood and persists in adulthood [1]. It is a heterogeneous condition that can include spastic (85%), dyskinetic (7%), which includes dystonia and choreoathetosis, and ataxic (4%) forms. CP motor severity can be established using the Gross Motor Function Classification System (GMFCS), which indicates a child’s level of gross motor function and mobility [2], ranking from I (light symptoms) to V (more severe). Children with CP are usually shorter and lighter than peers, especially when functional limitations are more severe [3,4]. The amount of body fat and muscle mass also tend to be lower, due to lack of mobility [5], as well as the duration and severity of the neurological disorder which reduces function in terms of daily activities [6]. There is a high risk of malnutrition (29–48%), which increases when motor impairment is higher [7,8,9]. Etiology of malnutrition is multifactorial and includes both nutritional and non-nutritional factors [10]. Dietary intake is conditioned by oromotor impairment [11] and the presence of gastroesophageal reflux and constipation [10]. Moreover, altered energy requirements frequently occur and decrease as ambulatory status declines and more limbs are involved [12]. Malnutrition impacts growth and quality of life [13], decreases immune function [4,14,15], increases use of health care [16], limits participation in social activities, and worsens survival prognosis [4,17]. In recent decades, it has been perceived that a correct diagnosis that leads to actions with the purpose of attenuating eating problems, decreases hospitalization rates and improves nutritional status [18]. This assessment will allow or adequate multidisciplinary interventions to restore linear growth, stabilize weight, reduce irritability and spasticity, improve circulation and healing, and increase social participation and quality of life [10]. There is no single method to assess nutritional status, but the analyses of a set of parameters can provide important nutritional information. In children, anthropometric parameters such as weight, height, and body mass index are the most frequently used since they are simple to collect and noninvasive [19]. For children with CP, anthropometric measurements can be difficult to assess, mainly if they have contractures, spasticity, scoliosis, and positioning problems that hinder the use of conventional weighing and other measuring methods [20]. In the last few decades, several indirect methods to determine anthropometrics in children with CP were developed, albeit with no consensus on the ideal one/ones despite de extensive discussion on the topic [20,21,22]. A variety of growth charts can be used to assess and monitor growth [23]. Specific charts for children with CP are available, such as Brooks charts [24]. However, they reflect how children with CP have grown, rather than how they can be expected to optimally grow [25]. National and other surveillance programs of children with CP provide clinical information [26,27] as well as nutritional status data. Countries that preform this analysis report elevated rates of undernutrition, but the most concerning is the higher underreport of anthropometric data [28,29]. To our knowledge, there is no record on the reasons why registers from the program do not always assess this evaluation parameters. The hypothesis was raised that registers experience some difficulties when assessing the nutritional status of these children. As part of a project funded by the Polytechnic Institute of Lisbon, IPL/2020/PIN-PC_ESTESL (Project for Nutritional Intervention in Cerebral Palsy), we developed a strategy to identify the main reasons for the higher underreport and developed actions to minimize this situation. This project included (1) assessment of registers’ motives for underreporting; (2) assessment of the difficulties experienced by healthcare professionals working with these children; (3) assessment of the feeding difficulties experienced by caregivers; and (4) a review of the literature on anthropometric procedures developed for children with CP and developed a pedagogical material adapted to the needs previous assessed.

## 2. Materials and Methods

### 2.1. Challenges While Registration Anthropometric Parameters: Questionnaire for Registers of Portuguese National Surveillance Program of Children with Cerebral Palsy

A questionnaire was developed and applied to a group of registers from a Portuguese national surveillance program of children with CP to access the major difficulties to fulfill nutritional information of national surveillance report. A cross-sectional survey was conducted using an original questionnaire developed by the research team and hosted by an online platform (Google Forms). The questionnaire was first applied to a group of healthcare professionals for piloted test to ensure functionality and clarity of questions. In August 2020, an invitation email was sent to 20 voluntary registers that follow children with CP within the scope of the national surveillance program. The survey was opened for five weeks. The anonymous questionnaire was focused on the following: importance given to the evaluation; existence of collaboration with other professionals to assess weight and height, and difficulties experienced when assessing (strategies that could facilitate those measurements). Data were aggregated descriptive analysis of categorical data was performed.

### 2.2. Challenges While Assessing Nutritional Status and Dealing with Feeding Problems: Meetings with Healthcare Professionals and Caregivers

A set of meetings were organized with healthcare professionals who work with children with CP—two in the hospital context and one in a CP center in a geographical area of Portugal (Alentejo). Quality data were recorded by one of the investigators, while two other investigators conducted the meeting. The meeting was structured in three phases. First, a presentation was made based on the nutritional data of the results of the last national report. Second, a collaborative brainstorming session was conducted where participants were able to freely share their beliefs and practical experiences. This allowed the research team to identify possible explanations for the higher underreporting results. Third, a workshop was held on conventional and alternative methodologies of anthropometric measurements for nutritional assessment with moments or practical assessment of children with CP where conventional and alternative anthropometric measurements were applied. Regarding the measured children, the caregiver was informed before the meeting about the anthropometric procedures and gave the informed consent to perform the evaluation with those healthcare professionals. Caregivers were also present during assessment. All of the procedures were explained, and the data were then included in the clinical record of the children. The ethical principles of the Helsinki declaration were followed. Another set of meetings was developed with caregivers. The quality data were recorded by one of the investigators while two other investigators conducted the meeting. The meeting was structured in two phases. First, the project was explained and the importance of adequate food for the health of children with CP. In the second phase, an inquiry was carried out on the main difficulties associated with feeding these children. A participatory methodology was used in which participants had the possibility to freely share their experiences, beliefs, and expectations. Finally, the main shared difficulties were presented and, together, caregivers’ practical interventions to solve these problems were systematized.

### 2.3. Development of the Toolkit for Healthcare Professionals and Registers

A narrative review of the literature was made following the SANRA criteria [30]. The search using PubMed platform from 1980 and 2021 was performed with the following search terms ‘cerebral palsy’, ‘children with disabilities’; ‘neurological disorders’; ‘nutritional status’; ‘body composition’; ‘body weight’; “weights and measures”, ‘’child development’; ‘physical examination’; ‘growth disorders’; ‘anthropometry/methods’; ‘assessment, nutritional’; ‘feeding problems’; ‘constipation’ and ‘gastroesophageal reflux’. For initial exploratory research on the topic, only systematic review articles and meta-analysis were considered. Articles in English, Portuguese and Spanish were included. The purpose was to investigate conventional and alternative methods of evaluating anthropometric parameters and nutritional status in children with CP, as well as common feeding problems. The articles cited in those were investigated in more detail, especially when they indicated descriptive procedures of measuring these children and how to interpret the results. The assessment methods to estimate weight and height recommended by ESPGHAN were chosen over the others, even though these were also considered as alternatives. After analysis of all of the data collected, two investigators with a communication background elaborated a practical toolkit to help healthcare professionals in nutritional assessment (Supplementary Material) and other to assist caregivers on feeding problems (data not shown). To illustrate the anthropometric procedures, original figures were created.

## 3. Results

### 3.1. Difficulties Experienced by Registers of the Surveillance Program and Healthcare Professionals When Assessing Nutritional Status and Suggested Solutions

Of the 20 registers of the Portuguese national surveillance program of children with CP invited to fulfil the questionnaire, 65% (*n* = 13) submitted their response. Most were medical doctors (*n* = 8), followed by physical therapists (*n* = 2), dietitians (*n* = 2), and nurses (*n* = 1). All of them considered nutritional assessment important, with a focus on weight assessment (WA) and height assessment (HA). Regarding this matter, the difficulties revealed by these professionals were lack of time (15% for WA and HA), lack of collaboration with other health professionals (7% for WA and 23% for HA), child’s motor impairment (30% for both) and lack of equipment such as scale or stadiometer (38% for both). All of the inquired mentioned having a measuring tape in their work setting. Alternative methods, such as segmental length measures, were known by 69%. In regards of possible solutions to facilitate this assessment, 15% of the registers found brochures/flyers a useful tool to compile information about different methodologies, 84% suggested to create and design a guide/manual of procedures described step-by-step and 46% suggested a mobile app. It was mentioned by 92% that professional training about alternative methods of assessing nutritional status in children with high motor impairment would be helpful. After each meeting with the healthcare professionals, all perspectives from the 35 participants were recorded from an informal conversation. The group included general practitioners, pediatricians, speech therapists, physiotherapists, occupational therapists, psychologists, nurses, dietitians, and caregivers. The contributions from healthcare professionals were similar to those expressed in the questionnaire filled by registers. In order to remove some of the barriers to the assessment of nutritional status, they mentioned the creation of an online platform with all of the equations involved in nutritional assessment, where data can be inserted and automatically calculate the measurements; one can create an educational program for healthcare professionals; and one can gather a team of high skilled professionals that visits all CP centers in the country to evaluate and register all children. It was also mentioned that involving the community surrounding children with CP would be helpful. The goal was to promote an active contribution to the evaluation and registration of their anthropometric parameters. All difficulties experienced by registers and healthcare professionals, as well as the suggested solutions to improve the nutritional assessment are summarized in Table 1.

### 3.2. Difficulties Experienced by Caregivers When Dealing with Children’s Feeding Problems and Discussed Strategies to Minimize These Problems

During the meetings with caregivers, the main problems referred were the difficulty in meal’s preparation with adapted textures that meet nutritional needs; difficulty to ensure correct hydration status; fear of meals where fish is the main protein source due to presence of fish bones; and how to position the child during mealtime. These answers were recorded while an informal conversation was happening. Some strategies were discussed, including the instruction of the canteen staff in how to prepare different textured and nutritional meals and the creation of a manual with this information summarized; creation of a manual with information on ways to aromatize water and tips to encourage the consumption of liquids (e.g., alarms during the day or mark the bottles to control the intake during the day); and ways to deal with common gastrointestinal problems such as constipation, dysphagia and gastroesophageal reflux. In addition, it was mentioned that there was little financial support for the prescription of artificial nutrition for families with children with CP.

### 3.3. Toolkit to Healthcare Professionals and Registers

The toolkit for healthcare professionals and registers focused on methods to assess weight and height in children with CP since these are the only indicators currently asked in the surveillance program forms regarding the evaluation of nutritional status.

#### 3.3.1. Weight Assessment

**Figure 1:**
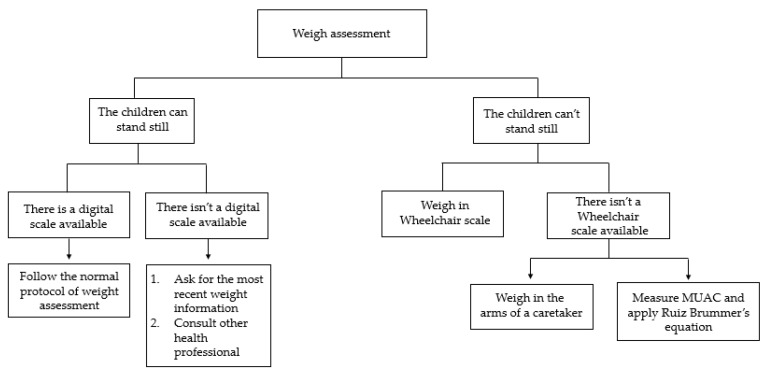
Weight assessment using conventional and alternative methods.

This can be performed directly on a scale or estimated by indirect measurement. A summary of these methods is described in Figure 1 and step by step procedures will be addressed ahead (Supplementary Material).

#### 3.3.2. Height Assessment

**Figure 2:**
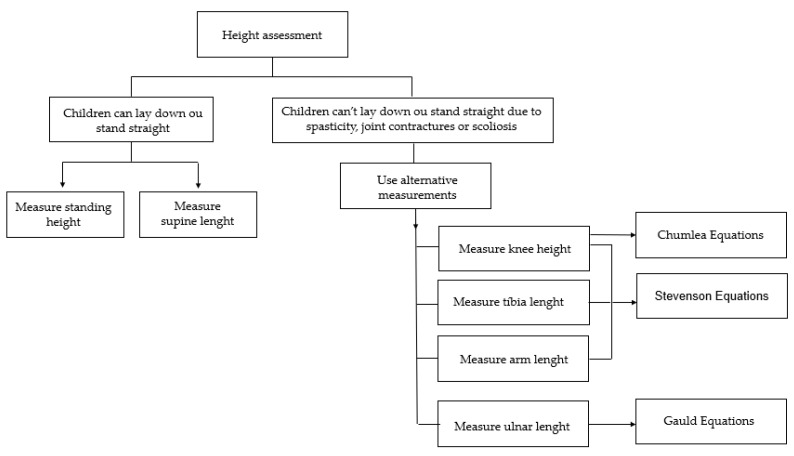
Height assessment using conventional and alternative methods.

As with weight, this can be performed directly with a stadiometer or estimated by indirect measurement. The summary of these methods is described in Figure 2, and the step-by-step methodologies will be addressed ahead (Supplementary Material).

Alternative methods may be needed to evaluate weight and height. These methods include the measurement of segmental lengths. The results are then included in equations and give us an estimation of these anthropometric parameters. In Table 2, these equations are summarized. The step-by-step description to assist the healthcare professional to perform each measurement according to the children characteristics and allow the calculation of the child’s weight and the height is presented in detail in the toolkit (Supplementary Material). The second toolkit dedicated to help caregivers included practical ways of dealing with the need of adapt meal textures, gastroesophageal reflux, constipation, nutritional deficiencies, and dehydration (data not shown).

## 4. Discussion

The work fields of the healthcare professionals included in this study were wide for both registers and healthcare professionals that work with these children. These different healthcare settings are beneficial insofar as they allow more opportunities to evaluate these children so that the anthropometric information is always up to date [35].

Several difficulties were mentioned in assessing nutritional status in children with CP, including lack of time and collaborations with other colleagues, child’s motor impairment and lack of equipment. There seems to be little autonomy and knowledge regarding this evaluation in order to do it in a short amount of time during a routine appointment. Children’s motor impairment made it difficult to carry out the nutritional assessment using conventional methods [32]. Therefore, it is important that healthcare professionals are trained to apply alternative methods, such as measuring segmental lengths and using the results in specific equations. The lack of specialized equipment referred by the participants can be overcome with the use of an anthropometric tape (all of the professionals involved mentioned that they have one).

As for our knowledge, this is the first time the reasons behind the high underreport on anthropometric data from registers in the Surveillance Program of Children with Cerebral Palsy are addressed. High omission rates of weight and height information have been reported. In Portugal, the omission rates are 53% for weight and 62.3% for height [29]. In a study from Nepal, the nutritional status information in national reports was also very limited and the authors say that this gap in evidence remains a major obstacle for planning targeted nutritional intervention for children with CP [28]. Thus, it is important to promote ways to improve the availability of this information.

From the results from the questionnaire applied to registers, it was possible to understand that educational tools would be helpful. This includes the development of a manual which can serve as a theoretical basis to create informative brochures and other educational materials as online presentations on the topic. The healthcare professionals suggested in the meetings that the community could be more involved once there are a lot of people living alongside these children who can weight and measure them routinely. The creation of a skilled team to carry out this evaluation in each country or region could be a solution, but would be more challenging to implement in a short amount of time and would have no financial support. The development of an online platform that includes all of the equations used in nutritional assessment was also referred to. This tool would speed up the interpretation of the results measured during each appointment and could also include the step-by-step procedures addressed in the manual. The challenges shared by these professionals (registers of the surveillance program and the other healthcare professionals) are similar and the different solutions addressed can be applicable to both groups. The main goal is to have weight and height information available in order to have updated reports on epidemiological data and to do adequate interventions that improve these children’s lives. For the present work, the toolkit was created as a first strategy to reduce the underreport on the surveillance program. Moreover, the meetings with healthcare professionals were also an opportunity to educate on performing nutritional status evaluation. In the future, the other suggestions can be implemented.

Some of challenges addressed by the caregivers are known and have been previously published in scientific literature [36,37]. Feeding problems are prevalent in children with CP (21–58%) and some of them were discussed during the meetings [36]. Nevertheless, the main problems expressed by caregivers were related to very practical issues, such as meal preparations and how to adapt its textures, how to prepare nutritious food, and other issues. The difficulties and suggestions were an added value for our communication professionals to develop tools that help caregivers dealing with those challenges (data not shown).

The development of a toolkit was important to ensure that the information is aggregated in a practical way so that health professionals can have quick access and to help in the decision-making when it comes to the assessment of anthropometric measures, such as weight and height. Although there is evidence on the issue of assessing the nutritional status of children with CP, a step-by-step manual is needed. In addition, it has original figures that exemplify the measures described to make it easier to put into practice. The design of the toolkit was based on a participatory process with health professionals who work with children with CP in their work settings and caregivers [38,39]. Understanding their previous knowledge, beliefs and resources about the nutritional assessment and feeding care of children with CP was necessary. In the theoretical framework of the Behavior Change Process [40] communication for behavior change is urgent to understand at what stage of knowledge about a particular problem, or the need to adopt a new behavior people are. Therefore, it is essential to adapt messages to communication according to these restrictions. Studies suggest that individuals are more likely to change behavior when interventions are culturally appropriate, locally relevant, and participated in at all stages [41].

The present work focus on the evaluation of weight and height in children with CP due to the urgent need to improve the availability of data in the surveillance program reports. Evaluation methods used should be feasible, efficient, inexpensive, and non-stressful for the child and caregivers. Measurements should be performed repeatedly to prevent incorrect diagnoses and to monitor continuously [17]. Moreover, the same method should be applied in order to gain experience and to allow a proper evaluation of these children. In this toolkit, only the measurements of weight and height were addressed. In the future, it is important to perform a complete evaluation of these children’s nutritional status and to ask the children’s clinical history and search information on their activity level, including the presence of comorbidities and pharmacotherapy that may affect nutritional status [42]. ESPGHAN recommends assessment of anthropometrics, body composition, and laboratory parameters [43]. It is recommended that one looks for warning signs of undernutrition including pressure ulcers, weight for age/sex Z-score below −2, triceps skinfold thickness and arm circumference or arm muscle area for age/sex below 10th centile and grow or weight gain velocity impairment or difficulty in recovering [17].

Body mass index (BMI), weight for height, and ideal body weight are frequently used to estimate body fat or nutritional status but are poor predictors of body fat percentage and have limited usefulness in guiding nutritional intervention [44]. Skinfolds reflect body mass reserves and could start to be routinely performed in children with CP, even if in some cases it is a challenging task [45]. This type of assessment should be performed by an experienced healthcare professional so that measurements are accurate. Triceps and subscapular skinfolds can be measured using the common methodology. It should be taken into account that the results must be interpreted considering a more localized distribution of fat in the abdominal area [7,46]. The results can be then applied in specific equations to predict body fat mass percentage shown in Table 3. The Slaughter equation, used in a healthy population, is not suitable for children with neuromotor impairment. Instead, Gurka equation, which includes correction factors such as GMFCS classification, best correlates with gold standard method [47]. Anthropometric parameters should be performed on the least affected side of the body [47,48,49]. Bioelectrical impedance analysis (BIA) allows for assessment of body composition, this being easy to perform, non-invasive, and involving no discomfort for the child. When available, it is more preferable than anthropometric measurements (skinfolds and body perimeters) as it is more suitable for children with CP [42]. Even so, limitations must be consider; hydration status is quite heterogeneous during childhood and depends on age and gender, and the results may also be falsified by the frequent dehydration state of children with CP [20,50].

Calculations of mid-upper arm muscle and fat areas are based on measurements of the upper arm circumference and triceps skinfolds [51] using the equation in Table 4.

Lohman classification can be used to classify the percentage of body fast mass results. It was created with a large sample of healthy children and can be used considering low body fat mass (≤10% for boys and ≤15% for girls), fat mass adequate (10–25% for boys and 16–30% for girls) and excess fat mass (>25% for boys and >30% for women). Values >30% of FM in boys and >35% of fat mass in girls indicates a likely diagnosis of obesity. When the percentage of fat mass is less than 7% in boys and less than 15% in girls, probably they will have very low weight [52]. Frisancho’s tables also provide centiles to classify PCT. After mid-upper-arm muscle circumference and mid-upper-arm muscle calculation, values can also be interpreted according to Frisancho centiles, even though these were not validated for children with CP. Therefore, they should be evaluated with caution. In children with CP, muscle mass values may be underestimated [51]. This information could be included in a future expanded version of the toolkit to guide healthcare professionals in nutritional status assessment, as suggested by ESPGHAN. Therefore, the clinical information of each child would be more complete and, perhaps, become part of the surveillance program form.

## 5. Conclusions

The amount of time needed to perform all of the care to children with CP limits the time to carry out anthropometric measurements, especially if the procedures are not described in a perceivable way. The great variety of methodologies used to assess nutritional status in these children make the choice challenging. So, healthcare professionals and registers should be instructed on how to carry out the procedures, otherwise the underreport of weight and height information will still be rising. The toolkit was designed to overcome some of the difficulties expressed by healthcare professionals, some of them registers in the national surveillance program. Having the information in a step-by-step structure with original illustrations to exemplify the procedures will hopefully help them in the evaluation. In the future, other strategies can be implemented accordingly with their suggestions. Caregivers also experience some challenges when dealing with the feeding problems of these children. A toolkit was developed to help them improve the nutritional assessment of children with CP, identify cases of malnutrition and enable faster and more personalized nutritional intervention (data not shown). This kind of support will need to be continually updated to be in line with the latest recommendations.

## Figures and Tables

**Table 1:** Difficulties experienced by registers and healthcare professionals and suggest strategies to improve nutritional assessment.

Group	Difficulties Experienced	Suggested Solutions
Registers of the Surveillance Program	Lack of time Lack of collaboration with other health professionals Child’s motor impairment Lack of equipment such as scale or stadiometer	Educational brochures/flyers Step by step descriptive manual of procedures mobile app Professional training
Healthcare professionals present in the meetings		Professional training Online platform to calculate the all of the equations automatically Skilled team that evaluates children all over the country Involve the community in nutritional assessment

**Table 2:** Equations to estimate weight and height in children with CP.

Weight			
Author	Equations		Segmental Measures Needed
Brunner equation [31]	If GMFCS level I–III:	Estimated Weight = 2.52 × MUAC(cm) + 1.19 × age (years) − 32	MUAC—mid upper arm circumference
	If GMFCS level IV–V:	Estimated Weight = 2.02 × MUAC(cm) + 0.97 × age (years) − 22.5	
Height			
Stevenson [32]	Height = (4.35 × AL) + 21.8		Arm length (AL)
	Height = (3.26 × TL) + 30.8		Tibial length (TL)
	Height = (2.69 × KH) + 24.2		Knee height (KH)
Chumlea [33]	Caucasian boys	Height = 40.54 + (2.22 × KH)	Knee height
	African-American boys	Height = 39.60 + (2.18 × KH)	
	Caucasian girls	Height = 43.21 + (2.15 × KH)	
	African-American girls	Height = 46.59 + (2.02 × KH)	
Gauld [34]	Boys	Height = (4.605 × UL) + (1.308 × A) + 28.003	Ulna lenght (UL)
	Girls	Height = (4.459 ×UL) + (1.315 × A) + 31.485	

**Table 3:** Original Slaughter equations and corrections for children with CP.

Slaughter’s Equations	Sum (triceps, subscapular) ≤ 35 mm	Boys	TSC * 1 e 2 TSB * 1 e 2 TSC 3 TSB 3 TSB 4 e 5 TSB 4 e 5	% FM * = 1.21(TFS * + SUBF *) − 0.008(TFS + SUBF)^2^ − 1.7 % FM = 1.21(TFS + SUBF) − 0.008(TFS + SUBF)^2^ − 3.2 % FM = 1.21(TFS + SUBF) − 0.008(TFS + SUBF)^2^ − 3.4 % FM = 1.21(TFS + SUBF) − 0.008(TFS + SUBF)^2^ − 5.2 % FM = 1.21(TFS + SUBF) − 0.008(TFS + SUBF)^2^ −5.5 % FM = 1.21(TFS + SUBF) − 0.008(TFS + SUBF)^2^ − 6.8
		Girls		% FM = 1.33(TFS + SUBF) − 0.013(TFS + SUBF)^2^ − 2.5
	Sum (triceps, subscapular) > 35 mm	Boys		% FM = 0.783(TFS SUBF) + 1.6
		Girls		% FM = 0.546(TFS + SUBF) + 9.7
Gurka’s Equations [47]	Additional correction for	Overall correction Males More severe GMFCS * Black race Tanner stage 3 Tanner stage 4 e 5 Sum(triceps, subscapular) > 35 mm		+12.2 −5.0 +5.1 −3.1 +2.0 −4.6 −3.2

* TSC, tanner stage Caucasian; TSB, tanner stage black; TFS, triceps skinfold; FM, fat mass; SUBF, subscapular skinfold; GMFCS, gross motor unction classification system.

**Table 4:** Mid-upper arm muscle area.

Mid-upper arm muscle area	MMA * = (MUA C * (cm) − TFS * (mm) × 3.1416)^2^/(4 × 3.1416)

* MMA, upper arm muscle area; MUAC, mid-upper arm circumference; TFS, triceps skinfold.

## Data Availability

Not applicable.

## Supplementary Materials

The following are available online at https://www.mdpi.com/article/10.3390/nu14061180/s1, Supplementary Material: Toolkit: Weight and height assessment in children with cerebral palsy.
