# Supplementary material for: Embracing the Nutritional Assessment in Cerebral Palsy: A Toolkit for Healthcare Professionals for Daily Practice
Source: Nutrients. 2022 Mar 11;14(6):1180. doi: 10.3390/nu14061180 (PMC8950259; doi:10.3390/nu14061180)
Supplement: Supplementary file 1 [file nutrients-14-01180-s001.zip › nutrients-1610423-supplementary.pdf]

TOOLKIT

WEIGHT AND HEIGHT ASSESSMENT IN CHILDREN WITH CEREBRAL PALSY

PIN-PC
A NUTRIÇÃO E A ALIMENTAÇÃO NA PARALISIA CEREBRAL

FINANCIAL SUPPORT: IPL /2020 / PIN-PC_ESTESL

2022

WEIGHT AND HEIGHT ASSESSMENT IN CHILDREN WITH CEREBRAL PALSY

PIN-PC
A NUTRIÇÃO E A ALIMENTAÇÃO NA PARALISIA CEREBRAL

MALNUTRITION IN CHILDREN WITH CEREBRAL PALSY

THE IMPORTANCE TO ASSESS

Why?

- nutritional deficits;
- lack of physical activity;
- endocrine dysfunctions.

By deficit | Undernutrition

Cannot acquire necessary nutrients (e.g. eating difficulties)

Problems with oromotor function

By excess | Obesity

Difficulty in adapting physical activity to the degree of motor impairment; injuries and fractures.

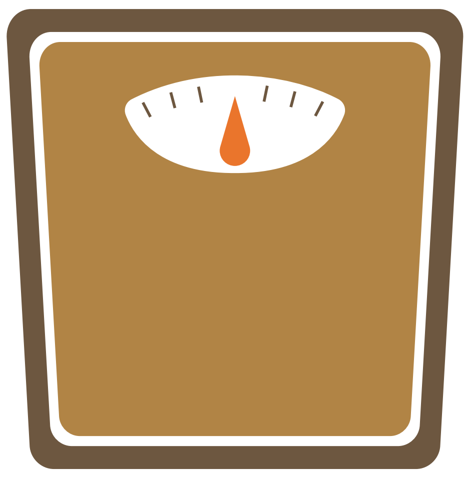

WEIGHT AND HEIGHT ASSESSMENT IN CHILDREN WITH CEREBRAL PALSY

PIN-PC
A NUTRIÇÃO E A ALIMENTAÇÃO NA PARALISIA CEREBRAL

SOMATOMETRIC DATA EVALUATION

MORE DIFFICULT WHEN THERE ARE CONTRACTURES, MOVEMENT DISORDERS OR MUSCULAR ATROPHY

WEIGHT

Children under 2 years of age: pediatric scale

Can remain standing and still during weighing

1. Check that the zero value is visible on the display when the scale is turned on;
2. Ask the child to be in light underclothes or the caregiver to undress him/her; If on diaper, ask to change to a dry one (and tare the diaper);
3. Record the value as quickly as possible to prevent the child from cooling down.
4. If the child has corrective prosthetics, subtract.

WEIGHT AND HEIGHT ASSESSMENT IN CHILDREN WITH CEREBRAL PALSY

PIN-PC
A NUTRIÇÃO E A ALIMENTAÇÃO NA PARALISIA CEREBRAL

SOMATOMETRIC DATA EVALUATION

MORE DIFFICULT WHEN THERE ARE CONTRACTURES, MOVEMENT DISORDERS OR MUSCULAR ATROPHY

Weight

Unable to stand still during weighing

Wheelchair-adapted scales are available

1. Check that the zero value is visible on the display when the scale is turned on;
2. Weigh the wheelchair without the child being seated;
3. Tare the scale with the wheelchair before placing the child in a sitting position;
4. Ask the child to be in light underclothes or the caregiver to undress him/her. If on diaper, ask to change to a dry one (and tare the diaper with the wheelchair);
5. Put the child in the wheelchair and record the value as quickly as possible to prevent the child from cooling down.
6. The weight of the wheelchair should be recorded for ease of procedure.

or

Wheelchair-adapted scales are not available

1. Use a trade scale;
2. Check that the zero value is visible on the display when the scale is turned on;
3. Ask the child to be in light underclothes or the caregiver to undress him/her
4. Ask the caregiver to remain still on the scale and tare the scale until the zero value reappears;
5. Place the child on the caregiver's lap so that the caregiver can hold him/her;
6. Record the weight of the child that appears on the display.

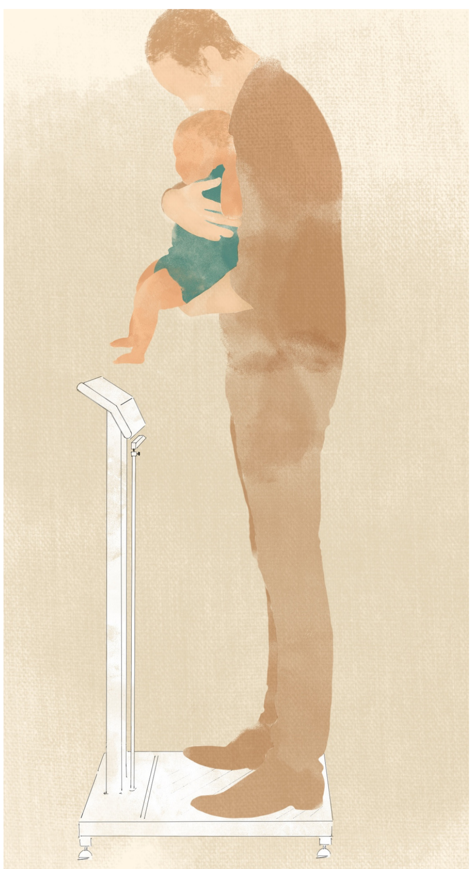

WEIGHT AND HEIGHT ASSESSMENT IN CHILDREN WITH CEREBRAL PALSY

PIN-PC
A NUTRIÇÃO E A ALIMENTAÇÃO NA PARALISIA CEREBRAL

MORE DIFFICULT WHEN THERE ARE CONTRACTURES, MOVEMENT DISORDERS OR MUSCULAR ATROPHY

SOMATOMETRIC DATA EVALUATION

Weight

Measure MUAC and apply Brunner's Equation

Measure mid-upper arm circunference

1. Select the less affected arm and undress it;
2. Bend the arm at 90° angle at the elbow, with the palm facing up and stretched out as much as possible;
3. Find acromion and olecranium and mark the midpoint with an anthropometric tape;
4. Measure arm circumference at marked point;

When	Equation to predict weight
GMFCS level I–III	2,52xMUAC(cm)+1,19x age (years) - 32
GMFCS level IV–V	2,02xMUAC(cm)+0,97x age (years) – 22,5

WEIGHT AND HEIGHT ASSESSMENT IN CHILDREN WITH CEREBRAL PALSY

PIN-PC
A NUTRIÇÃO E A ALIMENTAÇÃO NA PARALISIA CEREBRAL

SUPINE

STAND UP

SOMATOMETRIC DATA EVALUATION

HEIGHT

Without scoliosis, kyphosis or structural deformities in the flexion of the lower limbs

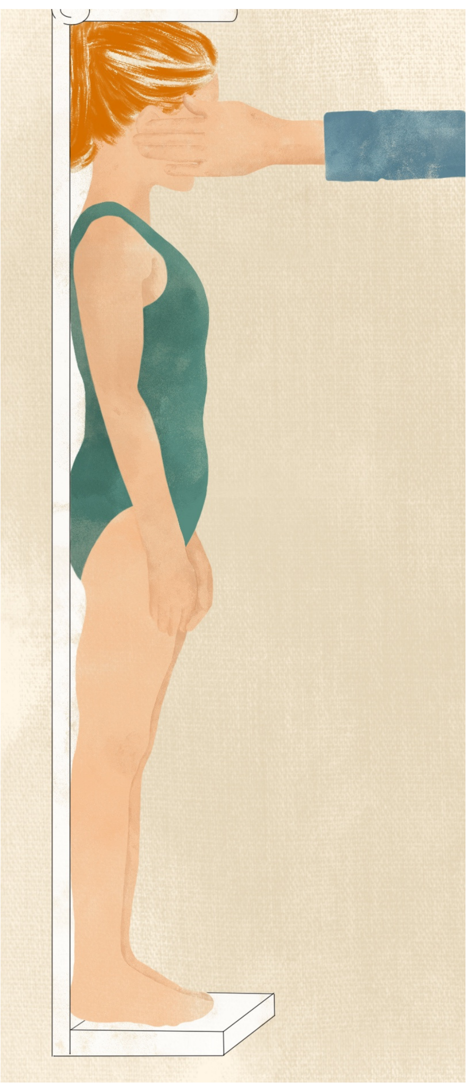

Stand up

Use a stadiometer

1. The assembled stadiometer must rest on a flat surface with maximum stability;
2. Ask the child and/or caregiver to take off their shoes and remove accessories that make the assessment difficult: hat, cap, bow.
3. Keep the child in an upright position, motionless, with arms extended along the body with the palms of the hands facing inwards;
4. Place the child's head in the Frankfurt horizontal plane and eyes looking to the ceiling
5. Place the child's heels and knees together and toes 60° apart;
6. Place the heels, the calf region, the pelvic girdle, the shoulder girdle and the occipital region in contact with the wall or the metal of the stadiometer in order to maintain balance;
7. Ask the child to inhale and hold the position;
8. Take the measurement with the stadiometer caliper at a 90° angle to the scale, moving it slowly until it compresses the hair and touches the highest point of the skull (vertex);

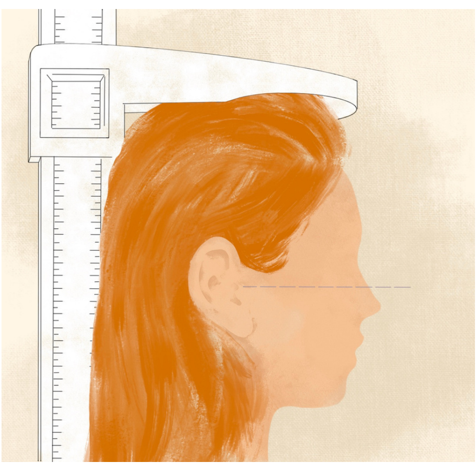

WEIGHT AND HEIGHT ASSESSMENT IN CHILDREN WITH CEREBRAL PALSY

PIN-PC
A NUTRIÇÃO E A ALIMENTAÇÃO NA PARALISIA CEREBRAL

SOMATOMETRIC DATA EVALUATION

SUPINE

STAND UP

HEIGHT

Without scoliosis, kyphosis or structural deformities in the flexion of the lower limbs

Supine	Use a caliper

1. Place the caliper on a smooth, not too soft and comfortable surface;
2. Cover the surface with a thin cloth or soft paper for hygiene and comfort of the child;
3. Lay the child in the supine position, with the head against the fixed plate, compressing the hair;
4. Ask the caregiver to stand behind the child to hold the child's head in position
5. Assure that the child remains in position so that the shoulder touches de caliper;
6. Hold the legs with one hand and apply light pressure to the knees to straighten them as much as possible without hurting them, while the other hand moves the movable plate against the soles of the child's feet. If it is not possible to keep both legs straight, measure only one and move the moving plate to the sole of the heel.

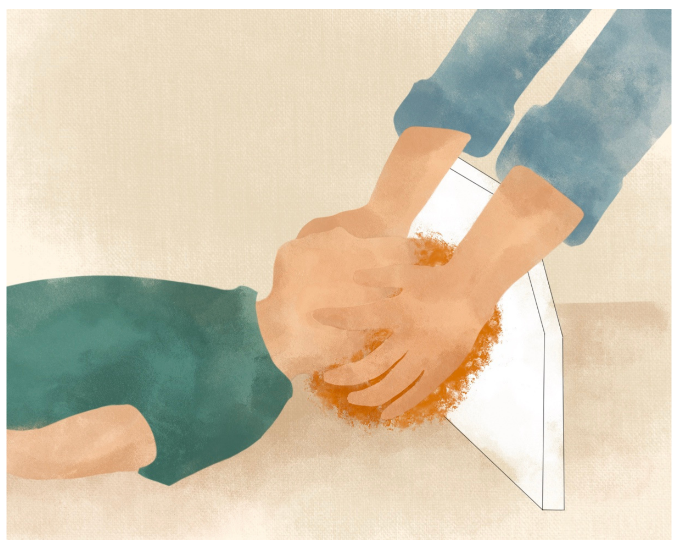

WEIGHT AND HEIGHT ASSESSMENT IN CHILDREN WITH CEREBRAL PALSY

PIN-PC
A NUTRIÇÃO E A ALIMENTAÇÃO NA PARALISIA CEREBRAL

SOMATOMETRIC DATA EVALUATION

SUPINE

STAND UP

Height

With scoliosis, kyphosis or structural deformities in the flexion of the lower limbs

<u>Alternative mesures</u> based on **body segment** measurement

Knee height
Length of the tibia
Arm length
Length of the ulna

ESPGHAN recommends measuring these segments and applying the values to the Stevenson Equation

Knee height

1. Place the child in a sitting position, naked from the knee down with barefoot;
2. Bend the left knee (or less involved side) and ankle in a 90° angle;
3. Use the caliper placing the fixed part positioned on the plantar surface of the foot (heel) and press the mobile part over the head of the patella (kneecap). The tape can be used instead of the caliper.
4. Apply the value obtained in the equation for this body segment.

Length of the tibia

1. Place the child in a sitting position, naked from the knee down, to expose measurement area;
2. Use an anthropometric tape to measure the length between the superomedial edge of the tibia and the lower edge of the malleolus;
3. Apply the value obtained in the equation for this body segment.

Segmental mesures	Equation to predict height
Knee height (KH)	(2.69 X KH) + 24.2
Tibia length (TL)	(3.26 X TL) + 30.8

WEIGHT AND HEIGHT ASSESSMENT IN CHILDREN WITH CEREBRAL PALSY

PIN-PC
A NUTRIÇÃO E A ALIMENTAÇÃO NA PARALISIA CEREBRAL

SUPINE
STAND UP

SOMATOMETRIC DATA EVALUATION

Height

With scoliosis, kyphosis or structural deformities in the flexion of the lower limbs

<u>Alternative mesures</u> based on **body segment** measurement

Knee height
Length of the tibia
Arm length
Length of the ulna

Knee height

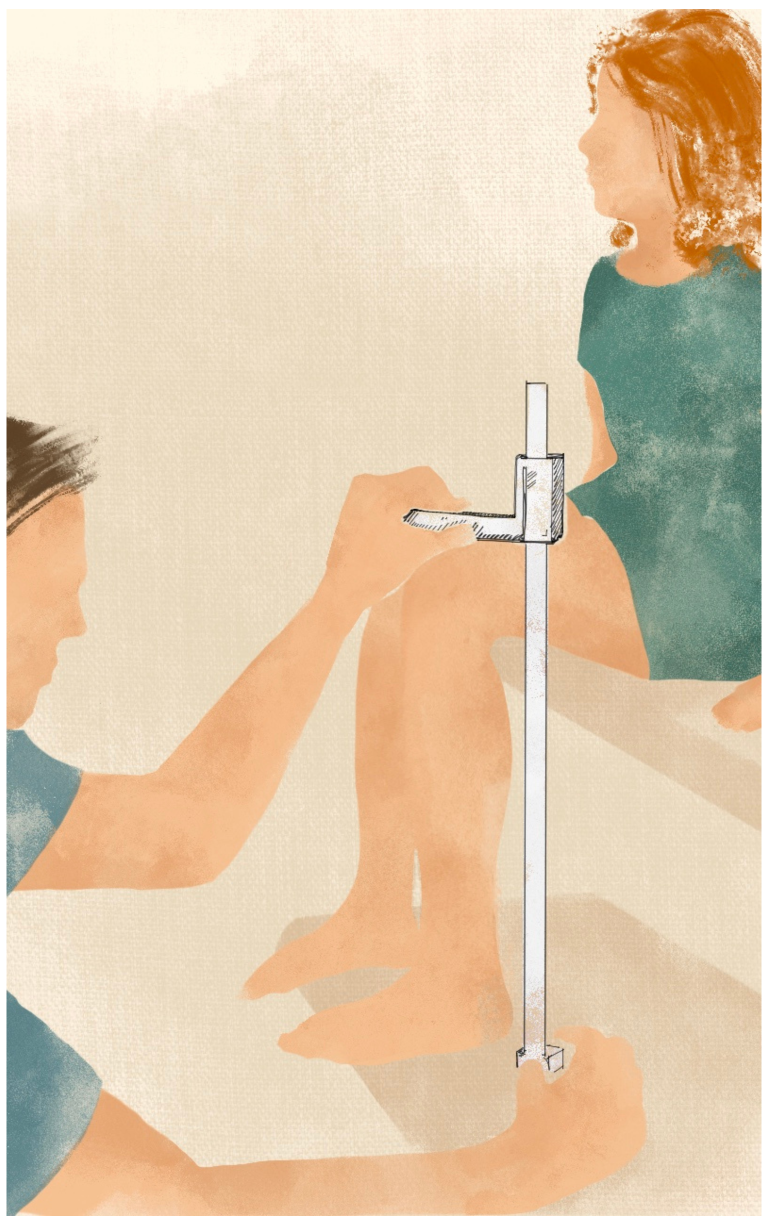

Length of the tibia

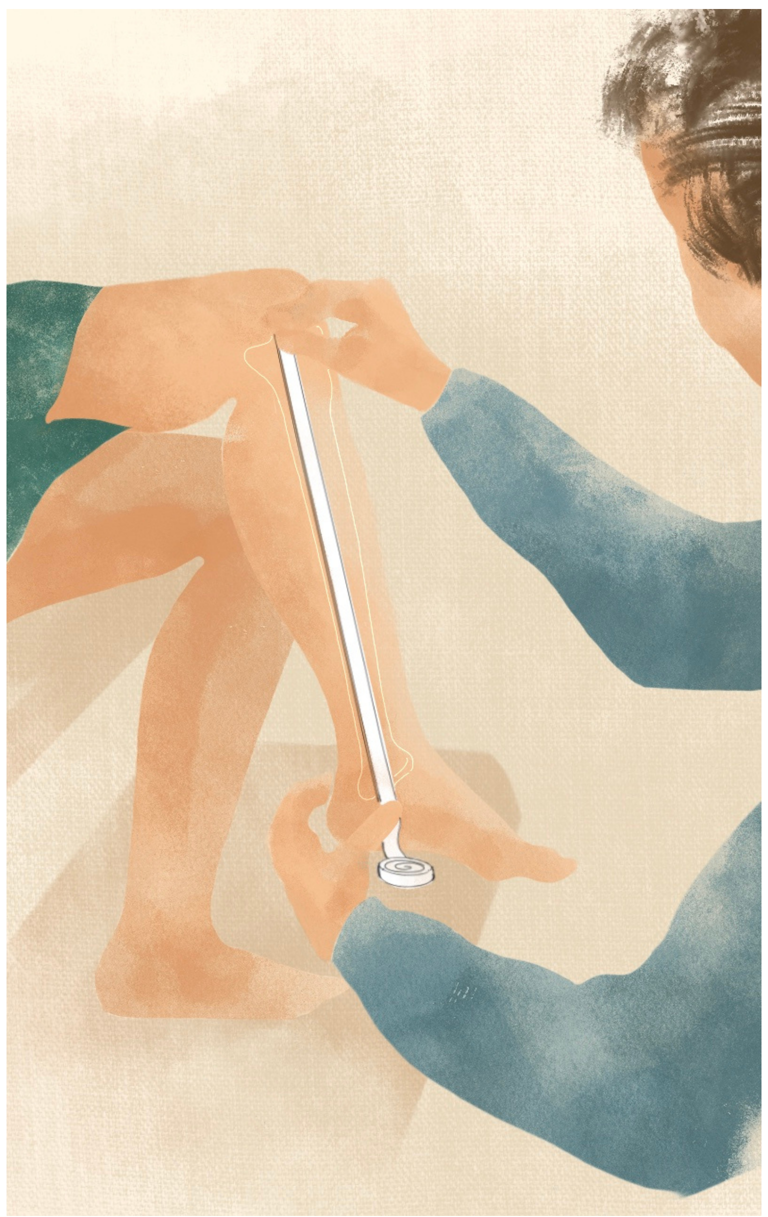

WEIGHT AND HEIGHT ASSESSMENT IN CHILDREN WITH CEREBRAL PALSY

PIN-PC
A NUTRIÇÃO E A ALIMENTAÇÃO NA PARALISIA CEREBRAL

SUPINE
STAND UP

SOMATOMETRIC DATA EVALUATION

Height

With scoliosis, kyphosis or structural deformities in the flexion of the lower limbs

Alternative mesures based on **body segment** measurement

Arm length --------- Apply Stevenson Equation

Length of the ulna --------- Apply Gauld Equation

Arm lenght

1. Place the child in an upright position, being able to be sitting or standing;
2. Position the child's arm in a relaxed manner, with the elbow at a 90° angle;
3. Use an anthropometric tape to measure acromion to radial head length;
4. Apply the value obtained in the equation for this body segment

Ulna lenght

1. Place the child in a sitting position with forearm resting on a table and the palm of the hand pointing downwards and the fingers straight. The elbow must be bent between 90° and 100°;
2. Palpate the ulna until it finds its proximal end and, in opposition, find the tip of the styloid process by palpating the bone distally until finding its end;
3. Use the anthropometric tape or an anthropometer to measure the distance between the two points found;
4. Apply the value obtained in the equation for this body segment

Segmental mesures	Equation to predict height
Arm lenght (AL)	$(4.35 \times AL) + 21.8$
Ulna length (UL) - boys	$(4.605 \times UL) + (1.308 \times A) + 28.003$
Ulna length (UL) - girls	$(4.459 \times UL) + (1.315 \times A) + 31.485$

WEIGHT AND HEIGHT ASSESSMENT IN CHILDREN WITH CEREBRAL PALSY

PIN-PC
A NUTRIÇÃO E A ALIMENTAÇÃO NA PARALISIA CEREBRAL

SUPINE
STAND UP

SOMATOMETRIC DATA EVALUATION

Height

With scoliosis, kyphosis or structural deformities in the flexion of the lower limbs

Alternative mesures based on **body segment** measurement

Arm length

Length of the ulna

Arm lenght

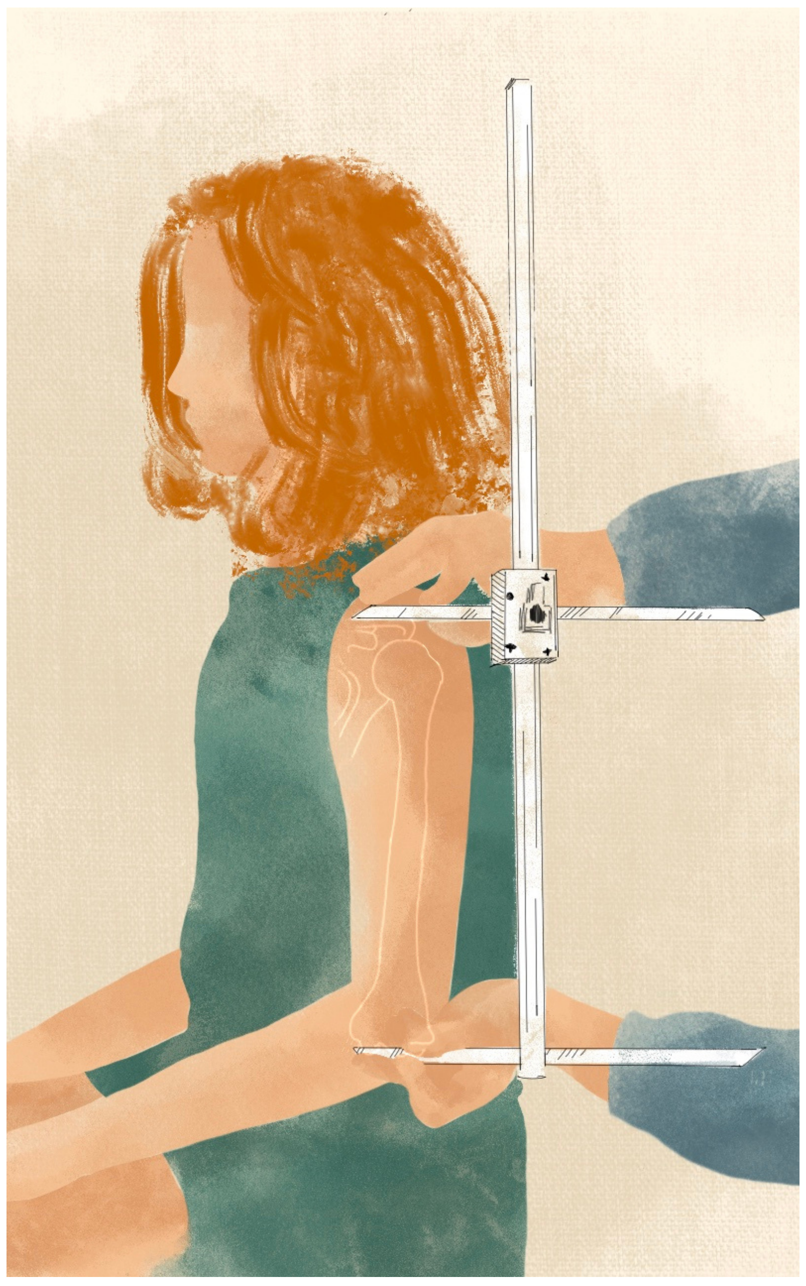

Ulna lenght

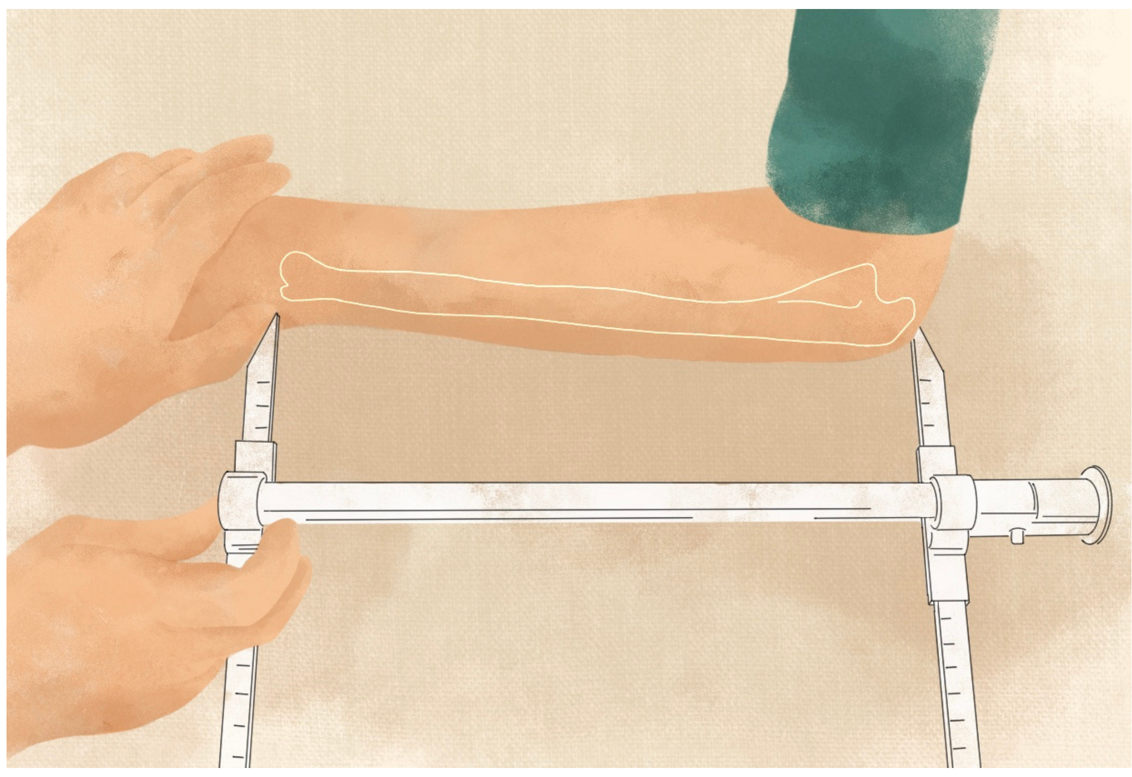

WEIGHT AND HEIGHT ASSESSMENT IN CHILDREN WITH CEREBRAL PALSY

PIN-PC
A NUTRIÇÃO E A ALIMENTAÇÃO NA PARALISIA CEREBRAL

NUTRITIONAL REGISTRATION PROCESS

NAME ____

GENDER Female__ Male__

BIRTH ____ / __ / __ **AGE** ______ **years**

WEIGHT

Weight (children) ___,__ Kg

Weight (children) + wheelchair + prostheses ___,__ Kg

Weight (wheelchair) ___,__ Kg

Weight (prostheses) ___,__ Kg

Weight (children) ______ Kg

HEIGHT

Height (children) ___ cm

Knee Height ___ cm — Height (children) ______ cm

Tibia length ___ cm — Height (children) ______ cm

Arm length ___ cm — Height (children) ______ cm

Ulna length ___ cm — Height (children) ______ cm
